# SECURE study: baseline findings from a UK prospective cohort of incidentally detected MGUS

**DOI:** 10.1038/s41408-026-01576-x

**Published:** 2026-07-20

**Authors:** Elizabeth Knight, Anupama Krishnamoorthi, Berrin Balik, Sulgi Byun, Richard Brouwer, Guy Pratt, Ross Sadler, Supratik Basu, Simon Stern, Stella Bowcock, Fenella Willis, Vicki Gamble, Aimee Maloney, Roderick Oakes, Grace Killingsworth, Patricia Nicholls, Karthik Ramasamy

**Affiliations:** 1 Medical Sciences Division, University of Oxford, Oxford, UK; 2 Oxford Translational Myeloma Centre, Oxford University Hospitals NHS Foundation Trust, London, UK; 3 Koc University Hospital, Istanbul, Turkey; 4 CRUK Clinical Trial Unit, University of Birmingham, Birmingham, UK; 5 The Royal Wolverhampton NHS Trust and University, Wolverhampton, UK; 6 Epsom & St Helier University Hospitals NHS Trust, London, UK; 7 King’s College Hospital NHS Trust, London, UK; 8 St George’s University Hospitals NHS Foundation Trust, London, UK; 9 North Cumbria Integrated Care NHS Foundation Trust, London, UK

**Keywords:** Epidemiology, Diagnosis, Haematological diseases

Dear Editor,

Monoclonal Gammopathy of Undetermined Significance (MGUS) is a clonal plasma-cell disorder characterised by the presence of a monoclonal protein without end-organ damage and is the earliest clinically detectable precursor to multiple myeloma (MM) [1]. MGUS is common in ageing populations, affecting 5% of adults over 50 years [2, 3]. Although all MM is preceded by MGUS, the average annual risk of progression is only ~1% [4, 5]. Progression endpoints differ by subtype: non-IgM MGUS typically progresses to MM, IgM MGUS often to lymphoplasmacytic lymphoma/Waldenström macroglobulinaemia, and light-chain MGUS to LC-myeloma or AL amyloidosis [6]. This long preclinical window makes risk communication and monitoring central to care.

Current International Myeloma Working Group (IMWG) guidance recommends periodic monitoring; however, prospective evidence demonstrating clinical benefit is limited and adherence in routine practice is variable [6, 7]. Beyond its role as a precursor, MGUS is associated with a range of comorbidities collectively termed Monoclonal Gammopathy of Clinical Significance (MGCS), including renal impairment and neuropathy, which contribute to morbidity and mortality [6, 8]. Importantly, most MGUS is detected incidentally during investigations for unrelated conditions, and up to 90% of patients who develop MM have no prior MGUS diagnosis [3, 9]. This highlights missed opportunities for earlier risk stratification and more consistent follow-up, while cautioning against resource-intensive population screening with uncertain clinical yield.

The SECURE study was established to address these information gaps by investigating a UK cohort of incidentally detected MGUS. It aims to generate a real-world reference for MGUS epidemiology, monitoring practices, and progression risk, including MGUS evolution, that can inform risk-adapted monitoring. SECURE integrates routine clinical data with patient-reported outcomes and serial biospecimens (immunology, proteomics, metabolomics, genomics), providing a platform to discover and validate biomarkers linked to progression, lived experience, and service utilisation.

SECURE is a UK, prospective, multicentre, non-interventional observational cohort of incidentally detected MGUS across multiple NHS sites (ClinicalTrials.gov NCT05539079; REC 22/WA/0291). Recruitment began September 2022 with planned sample size of 2000 and follow-up of up to 60 months per participant.

Eligibility criteria include adults (≥18) with confirmed MGUS by IMWG criteria (non-IgM, IgM, and light-chain subtypes). Exclusions include inability to consent, a borderline FLC ratio (0.3–3.0) in the absence of a measurable monoclonal protein on serum electrophoresis or immunofixation, and evidence of active/progressive plasma-cell disorder at inclusion. Patients are identified in routine care by haematology teams/specialist nurses/MDT coordinators; written consent follows ICH-GCP/Declaration of Helsinki. Optional consent covers biospecimen storage and linkage to primary/secondary care data for health-economics analyses.

Data collection at baseline and annually includes demographics, comorbidities/medications, laboratory parameters (M-protein, isotype), clinically indicated imaging, and patient-reported outcomes (questionnaires: EORTC QLQ-C30, PHQ-9, GAD-7, HAI, IUS, PCL-5). Data are captured securely in OpenClinica with planned linkage to HES/CPRD. At each visit, 10 mL serum and 10 mL EDTA are banked under SOPs at the Botnar Research Centre (with distribution to OUH Immunology, PC-B, and ICR for immunology/proteomics/metabolomics/genetics).

The primary outcome is progression from MGUS to MM. Secondary outcomes include routes to diagnosis (primary vs secondary care), monitoring patterns, family history, MGCS incidence, quality of life and psychological burden, and healthcare utilisation. Exploratory endpoints focus on biochemical, proteomics, and genomic predictors, such as metabolite signatures (sphingosine-1-phosphate, acetylcarnitine), post-translational modifications of serum free light-chains, and ctDNA dynamics, linked to progression.

To contextualise representativeness, ethnic distribution was benchmarked to ONS 2021 Census (England and Wales), and BMI categories to ONS 2022 survey data. Risk stratification followed Mayo Clinic criteria (non-IgG isotype, M protein ≥ 15 g/L, abnormal FLC ratio), with pathological FLC defined per updated iStopMM recommendations [10]. For this Correspondence, analyses are descriptive.

As of April 2026, 1244 individuals were recruited from 35 NHS sites. 1133 met inclusion criteria for this interim analysis (Fig. 1A). Median age was 71 years (range 25–95), and 53.0% were male (Table 1). Ethnicity was recorded for 1111 participants and plotted alongside ONS 2021 benchmarks (Fig. 1B). BMI was available for 970 participants, with category distribution shown alongside ONS 2022 benchmarks (Fig. 1C).

**Fig. 1:**
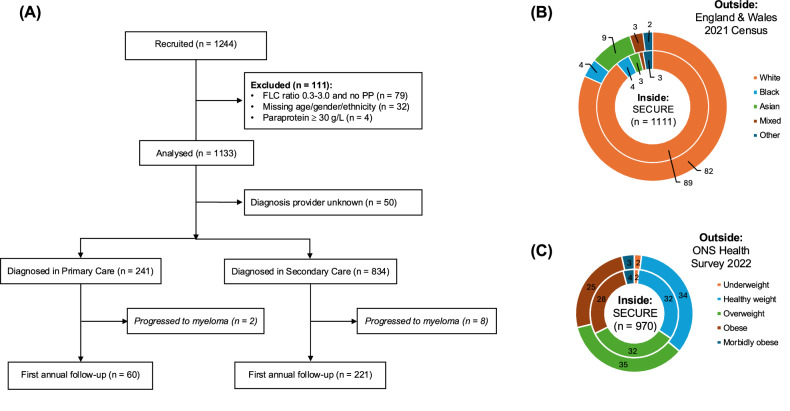
SECURE cohort recruitment flow and BMI/ethnicity benchmarking. **A** CONSORT-style recruitment and analysis flow with diagnosis provider breakdown. **B** Ethnicity distribution in SECURE versus England & Wales 2021 Census. **C** BMI categories in SECURE versus the ONS Health Survey 2022. Contains public sector information from the Office for National Statistics licensed under the Open Government Licence v3.0.

**Table 1:** Baseline characteristics of SECURE participants.

	*n*	%
Age (*n* = 1133)		
Median (range)	71 (25–95)	
20–29	2	0.2
30–39	12	1.1
40–49	41	3.6
50–59	168	14.8
60–69	304	26.8
70–79	447	39.5
80–89	151	13.3
≥90	8	0.7
Gender (*n* = 1133)		
Male	600	53.0
Female	533	47.0
Ethnicity (*n* = 1111)		
White	989	89.0
Black	47	4.2
Asian	33	3.0
Other	29	2.6
Mixed	13	1.2
BMI (*n* = 970)		
Underweight (<18.5)	16	1.6
Normal (18.5 ≤ *x* < 25)	312	32.2
Overweight (25 ≤ *x* < 30)	333	34.3
Obese (30 ≤ *x* < 40)	270	27.8
Morbidly obese (≥40)	39	4.0
Diagnosis route (*n* = 1083)		
Primary care	241	22.3
Secondary care	834	77.0
Other (e.g. private healthcare)	8	0.7
Risk stratification (*n* = 685)		
Low risk	207	30.2
Low-intermediate risk	307	44.8
High-intermediate risk	159	23.2
High risk	12	1.8
Progression risk factors (*n* = 685)		
Pathological FLC ratio	359	52.4
Non-IgG MGUS	205	29.9
M protein ≥ 15 g/L	97	14.2
Mean number of progression risk factors	0.97	
Comorbidities		
Autoimmune disease	110	16.3
Previous infection (with hospitalisation)	82	16.2
Osteoporosis	120	11.1
Family history (*n* = 972)		
First degree relative with FHx of MGUS/MM	44	4.5
First degree relative with FHx of other cancer	379	39.0

Risk stratification uses Mayo Clinic criteria, with the definition of pathological FLC informed by updated iStopMM recommendations. *BMI* body-mass index, *FLC* free light chain, *FHx* family history, *MGUS* monoclonal gammopathy of undetermined significance.

The route to diagnosis was available for 1083 participants: 22.3% via primary care and 77.0% via secondary care (Fig. 1A). Among those risk-stratified, 30.2% were low, 44.8% low-intermediate, 23.2% high-intermediate and 1.8% high risk for progression. We had 448 patients with missing risk factor data (majority paraprotein concentration) and therefore could not be risk stratified. Baseline progression factors among those with measurements: abnormal FLC ratio 52.4%, non-IgG MGUS 29.9%, M-protein ≥ 15 g/L 14.2% (Table 1). LC-MGUS prevalence was 7.1% (Supplementary 1).

Common comorbidities included autoimmune disease (16.3%, Supplementary 1), prior infection requiring hospitalisation (16.2%), and osteoporosis (11.1%). Relevant family history included 3.9% with first-degree MGUS/MM and 33.5% with first-degree cancer. To date, ten participants have progressed to MM. Time-to-event analyses are planned with mature follow-up.

Screening cohorts (e.g. iStopMM) answer questions about prevalence and screening logistics [11]. SECURE instead reflects how MGUS diagnoses are made and followed in clinics. In an iStopMM sub-study comparing screened to clinical (incidental) cohorts, the clinically detected group carried substantially more comorbidity than the screened group, despite broadly similar distributions of progression risk factors, and a slightly higher mean M-protein at baseline [12]. Complementing this, the Mayo Clinic comparison reported no meaningful difference in long-term progression between screened and clinically detected MGUS once death was treated as a competing risk. ‘Mode of detection’ did not add prognostic value beyond M-protein ≥ 15 g/L, non-IgG isotype, abnormal FLC ratio [13]. SECURE’s incidentally detected, clinic-based cohort in which MGCS and comorbidity are expected to be more frequent than in screened cohorts, with additional variables, can refine Mayo MGUS risk model. We intend to test age, sex, ethnicity and BMI, and develop risk-adapted follow-up strategy for incidental MGUS patients.

The median diagnosis age in SECURE is 71 years, similar to the clinical (non-screened) MGUS cohort from Mayo Clinic (72 years) and the iStopMM clinical cohort (73.5 years) [12, 13]. Although older age is often associated with higher biological risk of progression to MM [5], effect sizes vary across cohorts [14]. Given the slow natural history and non-myeloma mortality in older adults, the payoff from frequent surveillance is often small, arguing for risk-based follow-up. In SECURE, many patients carry at least one risk feature but relatively few formally classify as high progression risk (Table 1).

SECURE shows a slight male predominance (53.0%), like the clinical cohorts from Mayo (54%) and iStopMM (50.8%) [12, 13]. Prior literature on sex differences is varied, with some analyses suggesting lower progression risk in men, and others finding minimal effect size [5]. As such, sex should be considered as a contextual, not deterministic factor in risk-adapted pathways.

SECURE’s ethnicity distribution follows England & Wales 2021 Census (Fig. 1B), showing this incidental MGUS cohort is representative. Given reports of higher MGUS prevalence in people of African ancestry, a representative cohort helps plan monitoring practices.

SECURE’s BMI profile is similar to the ONS Health Survey distribution (Fig. 1C) and is consistent with reports linking adiposity to MGUS and, in some analyses, to progression [14]. While causality cannot be inferred from these interim data, the prevalence of overweight/obese individuals supports integrating weight management and bone-health optimisation into routine advice. With longitudinal follow-up, SECURE can explore whether changes in weight track with progression signals and prognosis.

Our observed comorbidity pattern (osteoporosis, autoimmune disease, serious infection) is consistent with prior links between MGUS and autoimmune/inflammatory conditions and infection. Some associations will reflect ascertainment (e.g. fracture or renal evaluations prompting protein studies), these patterns may nonetheless serve as cues for targeted case-finding. Data collection is ongoing regarding MGCS prevalence and form, with the aim to show how often MGCS emerges and who needs targeted workup.

Beyond the clinical dataset, SECURE’s key feature is serial biosampling at routine visits, letting us follow trajectories and capture pre-progression material (rare in most cohorts). This supports biomarker discovery for risk-adapted follow-up. Furthermore, early SECURE patient-reported outcomes suggest minimal psychological burden related to follow-up, supporting the feasibility of risk-based pathways [15].

As an interim descriptive analysis, some missing data is expected under standard-of-care practice. For 203 (17.9%) the paraprotein is confirmed on immunofixation but falls below the limit of quantification or is obscured by β-region overlap, reflecting quantification limitations rather than diagnostic uncertainty. Case capture and testing differ between sites; standardisation is ongoing, and sensitivity analyses will assess bias. This is a clinically detected cohort (mostly secondary care), so is not directly comparable with screened populations. Ethnicity and other subgroup analyses are currently underpowered. With short follow-up, progression events are few; time-to-event and multivariable analyses will follow as the dataset matures. Because MGUS-negative individuals were not captured as a comparator, observed associations may reflect indications for testing rather than MGUS-specific associations.

SECURE is the first UK prospective cohort of incidentally detected MGUS seen in routine care, generating new data for risk-adapted follow-up. With serial samples, patient-reported outcomes, and linked service data, SECURE is positioned to characterise how incidentally detected MGUS differs from screened cohorts, quantify MGCS burden and monitoring patterns in routine UK practice, and generate biomarker data to inform risk-adapted follow-up.

## Supplementary information

Supplementary Table S1
