# Supplementary material for: SECURE study: baseline findings from a UK prospective cohort of incidentally detected MGUS
Source: Blood Cancer J. 2026 Jul 20;16(1):119. doi: 10.1038/s41408-026-01576-x (PMC13385578; doi:10.1038/s41408-026-01576-x)
Supplement: Supplementary file 1 — Supplementary Table S1 [file 41408_2026_1576_MOESM1_ESM.docx]

**Supplementary Figures/Tables**

**Supplementary Table S1. MGUS subtype distribution, autoimmune disease, and family history breakdown at baseline.**

|  | **n** | **%** |
| --- | --- | --- |
| **MGUS Type** (n = 1018) | | |
| IgG | 617 | 60.6 |
| IgM | 167 | 16.4 |
| IgA | 154 | 15.1 |
| Kappa Light Chain | 53 | 5.2 |
| Lambda Light Chain | 27 | 2.7 |
| **Autoimmune Disease** (n = 674) | | |
| No autoimmune disease | 564 | 83.7 |
| Rheumatoid arthritis | 26 | 3.9 |
| Psoriatic arthritis/Psoriasis | 11 | 1.6 |
| Polymyalgia rheumatica | 8 | 1.2 |
| Inflammatory bowel disease | 5 | 0.7 |
| Sjogren’s syndrome | 5 | 0.7 |
| Coeliac disease | 4 | 0.6 |
| Lupus | 4 | 0.6 |
| Type 1 Diabetes | 3 | 0.4 |
| Autoimmune hepatitis | 3 | 0.4 |
| Graves’ disease | 2 | 0.3 |
| Vasculitis | 2 | 0.3 |
| Multiple sclerosis | 2 | 0.3 |
| Seronegative RA | 1 | 0.1 |
| Addison disease | 1 | 0.1 |
| Scleroderma | 1 | 0.1 |
| Other | 32 | 4.7 |
| **First Degree Relative Family History** (n = 416) | | |
| First degree relative with FHx of MGUS | 12 | 2.9 |
| First degree relative with FHx of Myeloma | 32 | 7.7 |
| First degree relative with FHx of other cancer | 379 | 91.1 |
| First degree relative with no FHx of MGUS/myeloma/other cancer | 9 | 2.2 |
